# Supplementary material for: Combining Experimental Evolution and Genomics to Understand How Seed Beetles Adapt to a Marginal Host Plant
Source: Genes (Basel). 2020 Apr 8;11(4):400. doi: 10.3390/genes11040400 (PMC7230198; doi:10.3390/genes11040400)
Supplement: Supplementary file 1 [file genes-11-00400-s001.pdf]

genes

MDPI

Article

Combining Experimental Evolution and Genomics to Understand HOW Seed Beetles Adapt to a Marginal Host Plant

Alexandre Rêgo [1,2,†], Samridhi Chaturvedi [3,†], Amy Springer [1], Alexandra M. Lish[1], Caroline L. Barton[1], Karen M. Kapheim [1], Frank J. Messina [1] and Zachariah Gompert [1,*]

1 Department of Biology, Utah State University, Logan, UT 84322, USA; alexandre.rego@aggiemail.usu.edu (A.R.); amy.springer@aggiemail.usu.edu (A.P.); alex.lish@aggiemail.usu.edu (A.M.L.); clbourgeois@hotmail.com (C.L.B.); karen.kapheim@usu.edu (K.M.K.); frank.messina@usu.edu (F.J.M.)
2 Department of Zoology, Stockholm University, 114 19 Stockholm, Sweden
3 Department of Organismic & Evolutionary Biology, Harvard University, Cambridge, MA 02138, USA; samridhi.chaturvedi@gmail.com
* Correspondence: zach.gompert@usu.edu
† These authors contributed equally to this work.

Received: date; Accepted: date; Published: date

Supplemental Tables & Figures

Table S1. Summary of data sets used in this study. Analyses and sample size for each *C. maculatus* line and generation are denoted. Gene expression samples comprise 30 larvae, with 15 reared on each host plant, and with sets of three larvae pooled for sequencing. Generation numbers for backcross (BC) mapping populations indicate the generation of the lentil line used to create the backcross. We have not maintained a generation counter on the south Indian line (M), but it has been in the lab for at least 300 generations.

Line	Generation	N	Var. calling	Change	Mapping	Expression
L1	F91	40	X			
ine L1	F100	40	X	X	X	
ine L1	F107	30				X
ine L1R	F35	40	X			
ine L1R	F46	40	X	X		
ine L1R	F55	30				X
ine BC-L1	F146	241	X		X	
ine L2	F78	40	X			
ine L2	F87	40	X	X	X	
ine L2R	F35	40	X			
ine L2R	F45	40	X	X	X	
ine BC-L2	F135	256	X		X	
ine L14	P	48	X	X		
ine L14	F1	48	X			
ine L14	F2	48	X			
ine L14	F3	48	X			
ine L14	F4	48	X			
ine L14A	F5	48	X			
ine L14A	F6	48	X			
ine L14A	F7	48	X			
ine L14A	F8	48	X			
ine L14A	F16	48	X	X	X	
ine L14B	F5	48	X			
ine L14B	F8	48	X			
ine L14B	F16	48	X			
ine BC-L14	F38	251	X		X	
ine M	n/a	48	X	X	X	
ine M	n/a	15				X

Table S2. Maximum likelihood estimates of hidden state transition probabilities from the HMM analyses. Here, states 1 and 2 denote the average and exceptional change states, and → indicates the direction of the transition.

Lines	1→1	1→2	2→1	2→2
M to L1 F100	0.996	0.004	0.764	0.236
M to L2 F87	0.996	0.004	0.759	0.241
L1 F91 to L1R F46	0.994	0.006	0.680	0.320
L2 F87 to L2R F45	0.995	0.005	0.654	0.346
L14 P to L14A F16	0.995	0.005	0.778	0.222

Table S3. REML estimates of the proportion of variation in adult weight and development time partitioned among families in each BC mapping population. The likelihood ratios (LR) and p-values from the null hypothesis test of no family variance are reported.

Line	Trait	Prop. var.	LR	p
BC-L1	Weight	0.074	10.418	0.0004
	Development Time	0.066	2.551	0.0374
BC-L2	Weight	0.074	2.169	0.0494
	Development Time	0.001	0.005	0.3733
BC-L14	Weight	0.097	11.113	0.0001
	Development Time	0.004	0.0443	0.3429

Table S4. Bayesian estimates of performance trait genetic architecture parameters, including the proportion of the trait variation explained by genetic effects (PVE), the proportion of the PVE attributable to genetic variants with measurable effects (PGE) and the number of causal variants or QTL (n_γ). Point estimates (posterior median) and 95% equal-tail probability intervals (ETPIs) are given.

Trait	Line	PVE	ETPI	PGE	ETPI	n_γ	ETPI
Weight	BC-L1	0.38	0.16, 0.61	0.26	0.00, 0.87	35	0, 260
Weight	BC-L2	0.14	0.02, 0.37	0.46	0.00, 0.94	11	0, 129
Weight	BC-L14	0.17	0.05, 0.33	0.30	0.00, 0.90	28	0, 215
Dev. time	BC-L1	0.08	0.01, 0.27	0.35	0.00, 0.92	16	0, 232
Dev. time	BC-L2	0.09	0.01, 0.24	0.40	0.00, 0.94	17	0, 224
Dev. time	BC-L14	0.08	0.01, 0.23	0.34	0.00, 0.92	16	0, 239

Table S5. Gene identifications for genes containing exceptional-change SNPs in more than one of the five comparisons. 'X' denotes comparisons where each gene contained exceptional-change SNPs. Comparisons are referred to by the second (derived) sample for each line and sample comparison (e.g., L1 F100 denotes change from M to L1 F100).

.

Gene	L1 F100	L2 F91	L1R F46	L2R F45	L14A F16
Armadillo repeat-containing protein 5	X	X			
ine Carbonic anhydrase 9	X	X			
ine Chromodomain-helicase-DNA-binding protein 9	X		X		
ine Cytochrome P450 4d2	X				X
ine Dynein heavy chain 7%2C axonemal	X				X
ine E3 ubiquitin-protein ligase MYCBP2	X				X
ine F-box/LRR-repeat protein 2			X	X	
ine Haloacid dehalogenase-like hydrolase domain-containing prot. 2	X				X
ine NADH dehydrogenase [ubiquinone] flavoprotein	X				X
ine Nuclear pore complex protein Nup155	X				X
ine Peptide-N(4)-(N-acetyl-beta-glucosaminyl)asparagine amidase	X	X			X
ine Synaptic vesicle membrane protein VAT-1 homolog-like	X	X			X
ine Transmembrane GTPase Marf	X				X
ine TWiK family of potassium channels protein 7	X	X			X
ine U3 small nucleolar RNA-associated protein 15 homolog	X				X

Table S6. Genetic correlations for adult weight breeding values estimated in each backcross mapping population based on the GWA mapping results from each pair of mapping populations. For example, the first row shows the genetic correlation for breeding value estimates of adult weight in BC-L1 given genetic SNP × weight associations in BC-L1 versus BC-L2. Pearson correlations, 95% confidence intervals (CIs) and associated *p*-values are reported.

Line	GWA	*r*	95% CI	*P*
L1	L1 × L2	-0.11	-0.22,-0.01	0.04
	L1 × L14	-0.01	-0.11,0.10	0.92
	L2 × L14	0.22	0.11,0.32	<0.01
L2	L1 × L2	-0.17	-0.27,-0.07	<0.01
	L1 × L14	-0.03	-0.14,0.07	0.56
	L2 × L14	-0.02	-0.13,0.08	0.67
L14	L1 × L2	0.05	-0.06,0.15	0.35
	L1 × L14	0.17	0.06,0.27	<0.01
	L2 × L14	-0.04	-0.15,0.06	0.42

Table S7. Genetic correlations for development time breeding values estimated in each backcross mapping population based on the GWA mapping results from each pair of mapping populations. For example, the first row shows the genetic correlation for breeding value estimates of development time in BC-L1 given genetic SNP × weight associations in BC-L1 versus BC-L2. Pearson correlations, 95% confidence intervals (CIs) and associated *P*-values are reported.

Line	GWA	*r*	95% CI	*P*
L1	L1 × L2	−0.25	−0.35,−0.15	<0.01
	L1 × L14	−0.17	−0.27,−0.06	<0.01
	L2 × L14	0.06	−0.05,0.17	0.27
L2	L1 × L2	−0.31	−0.41,−0.21	<0.01
	L1 × L14	−0.15	−0.25,−0.04	0.01
	L2 × L14	−0.04	−0.14,0.07	0.52
L14	L1 × L2	0.15	0.04,0.25	0.01
	L1 × L14	−0.07	−0.17,0.04	0.20
	L2 × L14	−0.16	−0.26,−0.06	<0.01

Table S8. Summary of differential expression of putative digestive enzymes. For each comparison, we report the number of differentially expressed proteases and carboxylases, and the binomial probability of having the observed number of each by chance. We classified genes as likely digestive proteases and carboxylases following [**?**]. protease = serine protease, trypsin, chymotrypsin, cathepsin, aspartic proteinase, lysosomal aspartic protease, cysteine protease or proteinase (88 genes); carboxylase = amylase, cellulase, glucosidase or maltase (29 genes). The first three comparisons correspond to genetic differences in expression, the next two to plastic (host) differences in expression, and the final four include genetic and plastic differences.

	**Protease**		**Carbohydrase**	
**Comparison**	**Obs.**	**P**	**Obs.**	**P**
$M^M \times L1^M$	3	0.07	0	0.70
$M^M \times L1R^M$	8	0.11	2	0.28
$L1^L \times L1R^L$	0	0.49	0	0.79
$L1^M \times L1^L$	0	0.81	0	0.93
$L1R^M \times L1R^L$	1	0.26	0	0.50
$M^M \times L1^L$	6	0.16	2	0.28
$M^M \times L1R^L$	7	0.11	4	0.06
$L1^M \times L1R^M$	0	0.91	0	0.97
$L1^L \times L1R^M$	0	0.70	1	0.10

Table S9. Summary of randomization tests results for the number of exceptional-change SNPs in M to L1 F100 or L1 F91 to L1R F46 among differentially expressed genes.

E&R	Dif. express.	Observed	Null	*P*
M→L1 F100	$L1^M \times L1^L$	3	0.243	0.017
	$LR1^M \times LR1^L$	4	2.180	0.203
	$L1^L \times L1R^L$	2	0.600	0.141
	$M^M \times L1^M$	3	1.124	0.138
	$L1^M \times L1R^M$	0	0.047	1.00
L1 F91→L1R F46	$L1^M \times L1^L$	0	0.295	1.00
	$LR1^M \times LR1^L$	1	2.185	0.801
	$L1^L \times L1R^L$	0	0.568	1.00
	$M^M \times L1^M$	9	1.090	1.00
	$L1^M \times L1R^M$	0	0.056	1.00

Table S10. Summary of randomization tests results for the density of adult weight and development time QTL (mean PIP across SNPs) within differentially expressed genes.

Trait	Dif. express.	Observed	Null	*P*
Weight	$L1^M \times L1^L$	0.0044	0.0044	0.4300
	$L1R^M \times L1R^L$	0.0044	0.0044	0.4740
	$L1^L \times L1R^L$	0.0044	0.0044	0.3500
	$M^M \times L1^M$	0.0043	0.0044	0.8230
	$L1^M \times L1R^M$	0.0046	0.0044	0.1100
Dev. time	$L1^M \times L1^L$	0.0033	0.0035	0.8170
	$L1R^M \times L1R^L$	0.0035	0.0035	0.4120
	$L1^L \times L1R^L$	0.0034	0.0035	0.7260
	$M^M \times L1^M$	0.0035	0.0035	0.3850
	$L1^M \times L1R^M$	0.0035	0.0035	0.2270

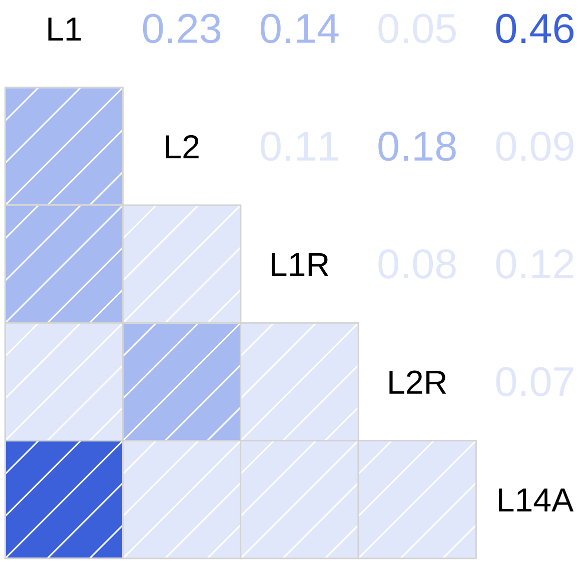

Figure S1. Correlogram summarizing correlations in evolutionary change across SNPs for pairs of lines. Names of the derived line in each pair are given along the diagonal. The direction and intensity (by shading) are depicted visually in the lower triangle, and the Pearson correlation coefficient for each pair of lines is given in the upper triangle

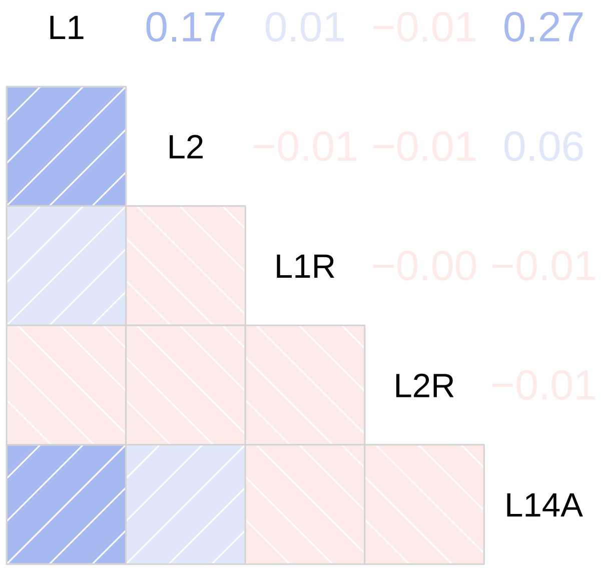

Figure S2. Correlogram summarizing correlations in hidden Markov model (HMM) state across SNPs for pairs of lines. HMM state refers to whether or not each SNP was classified as being in an exceptional change genetic region based on standardized allele frequency change. Names of the derived line in each pair are given along the diagonal. The direction and intensity (by shading) are depicted visually in the lower triangle, and the Pearson correlation coefficient for each pair of lines is given in the upper triangle.

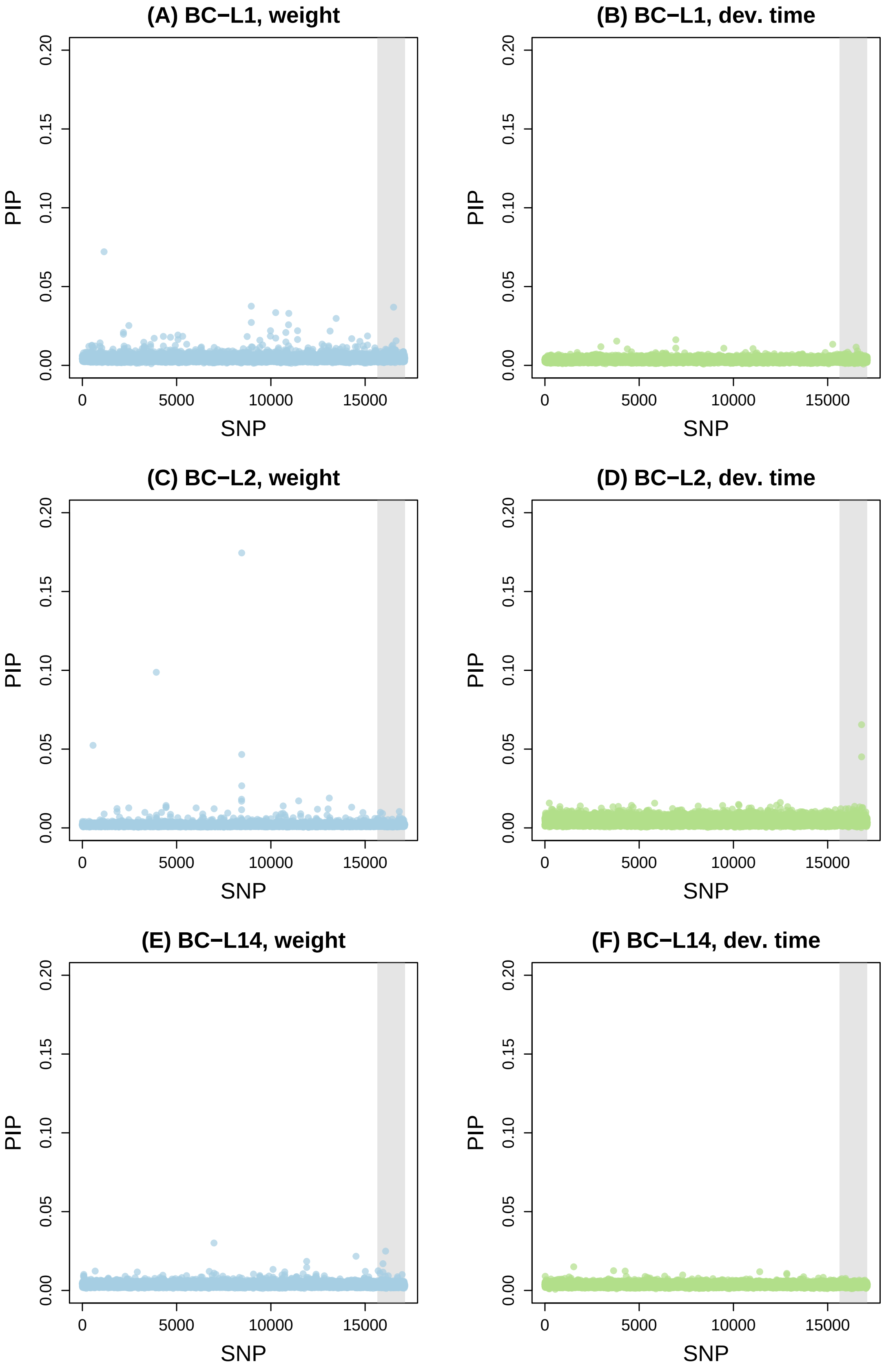

Figure S3. The Manhattan plots show posterior inclusions probabilities (PIPs) for SNPs for each back-cross line (BC-L1, BC-L2 and BC-L14) and trait (weight and development time). SNPs are ordered by scaffold and the gray shaded area denotes X-linked SNPs.

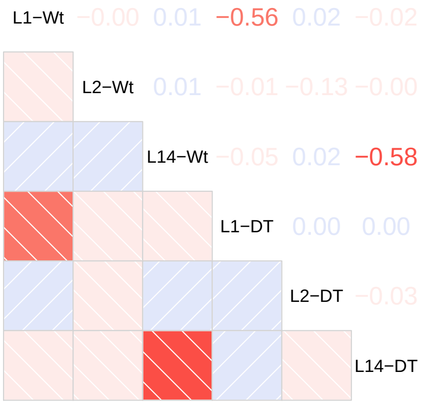

Figure S4. Correlogram summarizing correlations between model-averaged effect estimates for pairs of lines and traits. Back-cross lines (denoted L1, L2 and L14 for brevity) and traits (Wt = weight, DT = development time) are given along the diagonal. The direction and intensity (by shading) are depicted visually in the lower triangle, and the Pearson correlation coefficient for each pair of lines and traits is given in the upper triangle.

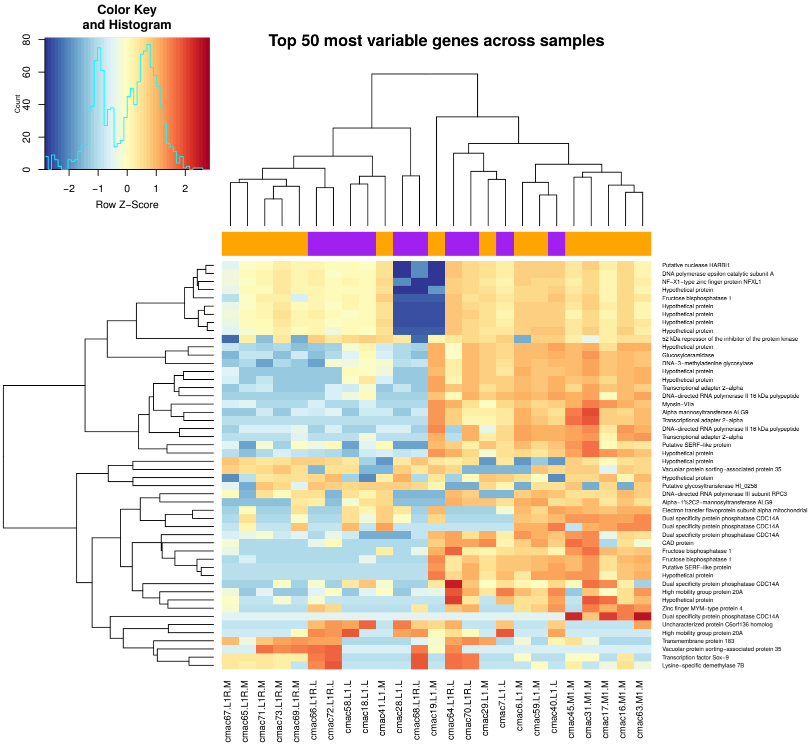

Figure S5. Hierarchical clustering and heatmap of the 50 most variably expressed genes across the entire data set for each sample. Sample IDs include a unique identifier for each pool of three larvae followed by the line (L1, L1R or M) and host treatement (L or M).

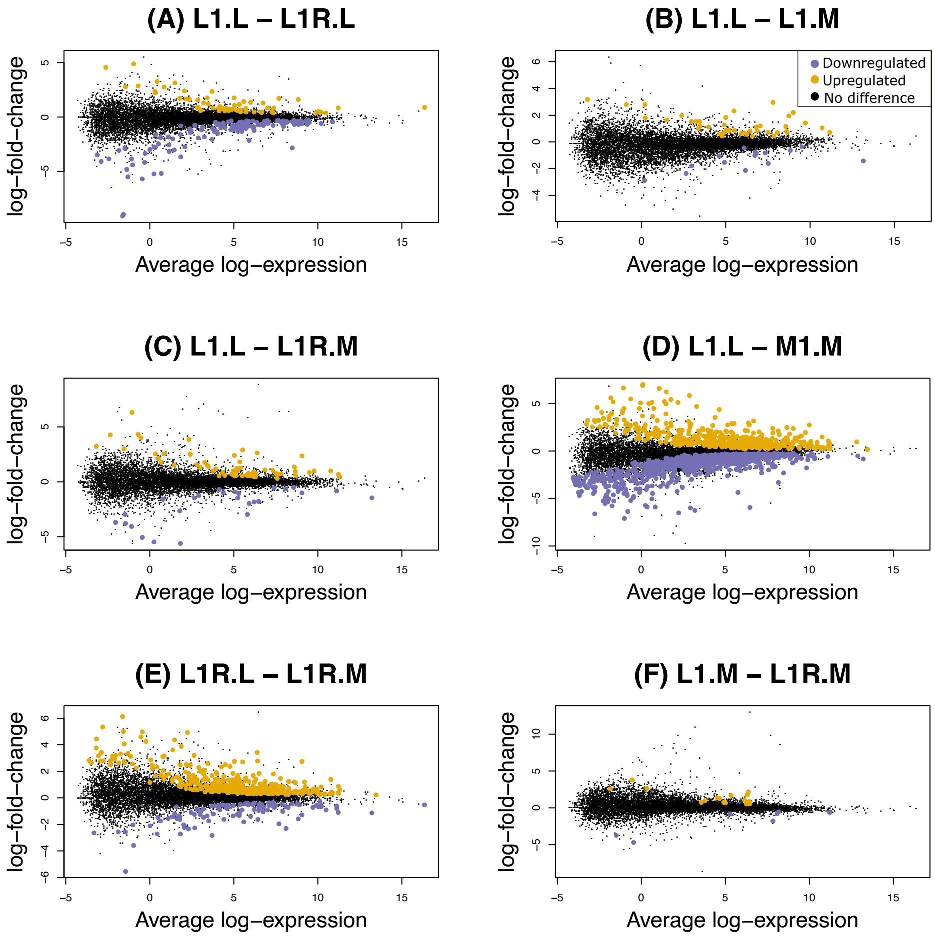

Figure S6. Mean differential expression plots for changes in gene expression between different pairs of contrasts. In the plot legends, 0 = genes that show no significant difference in expression between the contrast groups, 1 = genes that show significant upregulation in expression between the contrast groups,and −1 = genes that show significant downregulated in expression between the contrast groups. Each group is labeled by line and host.

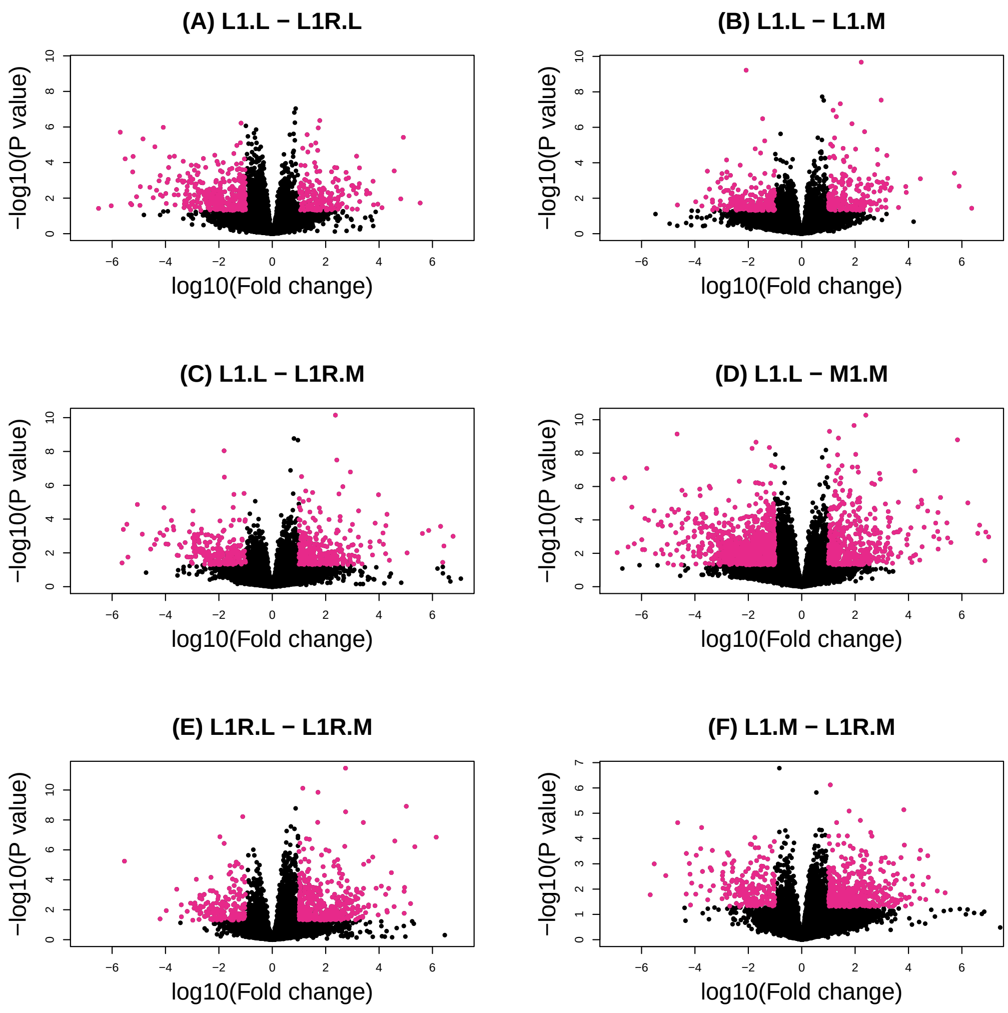

Figure S7. Volcano plots show gene expression ratios (log10 fold change) plotted against the negative transformed *P*-values generated from `decideTests` function from `voom` between different pairs of contrasts. Colored dots represent genes with a statistically significant ($P < 0.05$) fold change of > 1. Each group is labeled by line and host.

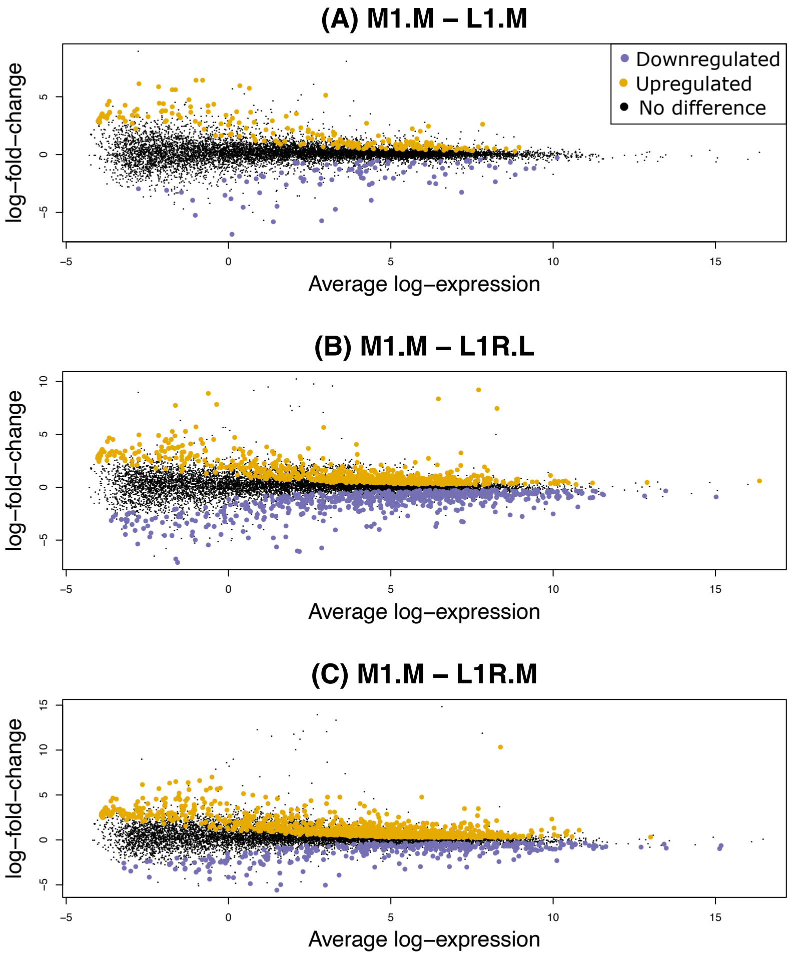

Figure S8. Mean differential expression plots for changes in gene expression between different pairs of contrasts. In the plot legends, 0 = genes that show no significant difference in expression between the contrast groups, 1 = genes that show significant upregulation in expression between the contrast groups,and −1 = genes that show significant downregulated in expression between the contrast groups. Each group is labeled by line and host.

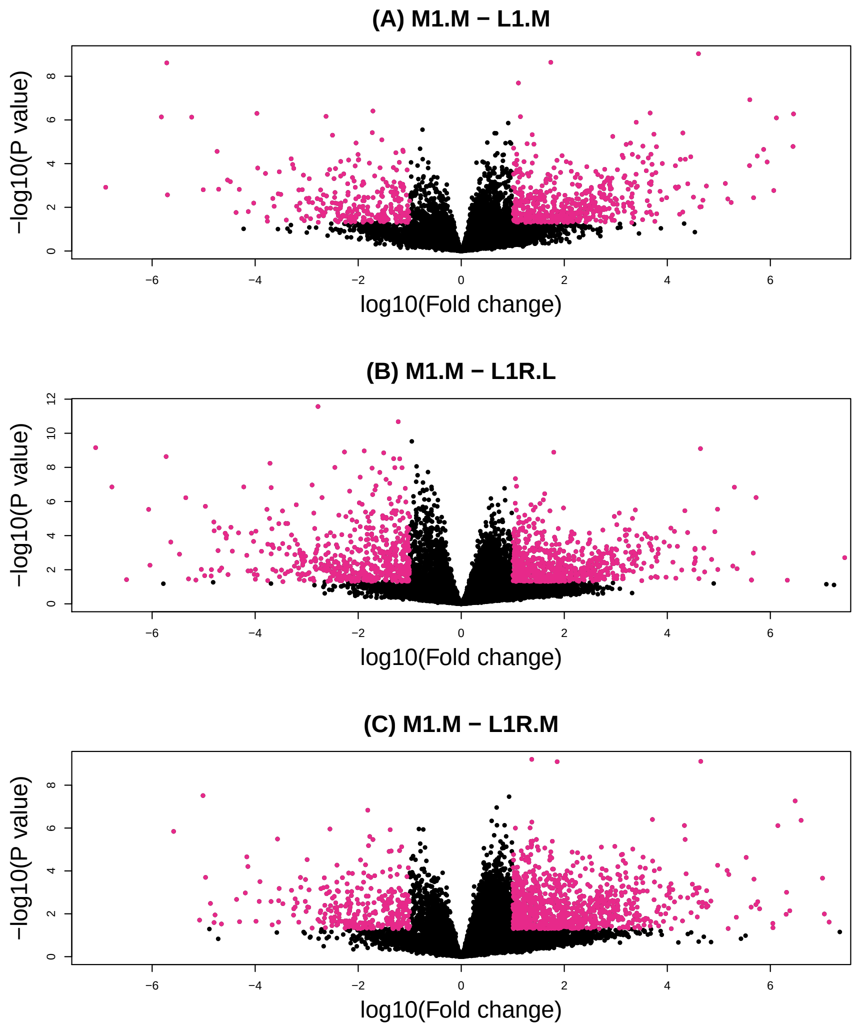

Figure S9. Volcano plots show gene expression ratios (log10 fold change) plotted against the negative transformed *P*-values generated from `decideTests` function from `voom` between different pairs of contrasts. Colored dots represent genes with a statistically significant ($P < 0.05$) fold change of > 1. Each group is labeled by line and host.

© 2020 by the authors. Licensee MDPI, Basel, Switzerland. This article is an open access article distributed under the terms and conditions of the Creative Commons Attribution (CC BY) license (http://creativecommons.org/licenses/by/4.0/).
